# Improving the sustainability of biodiesel by using imidazolium-based ionic liquid

**DOI:** 10.1038/s41598-021-96358-9

**Published:** 2021-08-19

**Authors:** M. A. Deyab, Q. Mohsen

**Affiliations:** 1 Egyptian Petroleum Research Institute (EPRI), Nasr City, Cairo, Egypt; 2 Department of Chemistry, College of Sciences, Taif University, Taif, Saudi Arabia

**Keywords:** Chemistry, Energy science and technology, Renewable energy

## Abstract

Corrosion of biodiesel-filled fuel tanks has become a major problem in the use of biodiesel as a new green energy source. The ionic liquid 1-Hexyl-3-methylimidazolium bis(trifluoromethanesulfonyl)imide [C_10_H_19_N_2_]^+^[C_2_F_6_NO_4_S_2_]^−^ was used to control corrosion of C-steel in non-edible biodiesel to resolve this problem. The anti-corrosion and antioxidant properties of the [C_10_H_19_N_2_]^+^[C_2_F_6_NO_4_S_2_]^−^ were characterized using weight loss, electrochemical impedance spectroscopy, total acid number measurements beside SEM and EDX analysis. The findings show that [C_10_H_19_N_2_]^+^[C_2_F_6_NO_4_S_2_]^−^ plays an important role in preventing C-steel corrosion in biodiesel with an efficiency close to 99 percent. The adsorption capability and antioxidant properties of [C_10_H_19_N_2_]^+^[C_2_F_6_NO_4_S_2_]^−^ are the major contributors to the ionic liquid's anti-corrosion properties. We anticipate that this work will help to sustainable expand the use of biodiesel as a renewable energy source.

## Introduction

Biodiesel is one of the most promising renewable energy sources of the future^1,2^. It can be used in the current diesel car engines without needing any changes to be made to them^3^. Principally, biodiesel is synthesized by trans-esterification process of vegetable oil with alcohol in the presence of catalyst^4^. The advantage of biodiesel over fossil fuels is that it is a clean fuel and does not cause pollution to the environment. In addition, it can be produced from non-exhaustible natural sources^5^. One of the most important obstacles that limit the widespread use of biodiesel is that it causes corrosion of fuel tanks^6,7^.

Several previous studies have shown that the rate of corrosion in the fuel tank containing biodiesel is much higher than that containing petrodiesel.

This is due to many factors such as hygroscopic nature of biodiesel and biodiesel oxidation^8^. This leads to the presence of water and free fatty acids in the fuel tank and consequently the corrosion in the wall of tank^9,10^. There are two methods that could be used to prevent the corrosion problem caused by the biodiesel namely: (i) use of high corrosion resistance alloys in the manufacture of fuel tank, and (ii) use of corrosion inhibitors to protect the fuel tank. High corrosion resistance alloys are expensive and require special manufacturing capabilities when used in the manufacture of fuel tanks^11,12^. In this regard, some organic compounds, surfactants and plant extracts were used to protect the fuel tank from corrosion in biodiesel. Cardanol^13^, rosemary leaves^14^, span 80^15^, and butylated hydroxyl toluene^16^ were found to protect the biodiesel fuel tank from corrosion in previous our studies. In this paper, we continue our research in this area by using a new class of compounds, ionic liquids, as a new additive in non-edible Neem oil biodiesel to prevent corrosion in the fuel tank.

Ionic liquids as corrosion inhibitors should be recommended over conventional volatile and toxic corrosion inhibitors^17–19^ because of their numerous advantages, including low volatility, chemical and electrochemical stability, and the possibility of being more environmentally friendly. Furthermore, ionic liquids are frequently used in smaller quantities than conventional corrosion inhibitors, leading to reduced cost.

For the first time, the effect of 1-hexyl-3-methylimidazolium bis(trifluoromethanesulfonyl)imide [C_10_H_19_N_2_]^+^[C_2_F_6_NO_4_S_2_]^−^ on the corrosion behavior of C-steel in biodiesel was investigated . The corrosion mechanism is also explained through adsorption isotherm and thermodynamic studies.

## Materials and methods

### Materials

The C-steel (composition wt%: 0.2 carbon, 0.6 manganese, 0.1 Silicon, balance Fe) substrate was purchased from Egyptian iron co. ASTM G1—03(2017)e1 standard method was used to produce clean C-steel electrode surface^20,21^.

The ionic liquid [C_10_H_19_N_2_]^+^[C_2_F_6_NO_4_S_2_]^−^ was purchased from Sigma-Aldrich Chemie GmbH.

Non-edible Neem oil was obtained from a local plant company with the following fatty acid profile: Palmitic acid (C16:0) (28.42%), Stearic acid (C18:0) (19.22%), Oleic acid (C18:1) (31.66%), Linoleic acid (C18:2) (19.40%), Arachidic acid (C20:0) (1.3%).

The synthesis of biodiesel was conducted in a conical flask containing 50 ml Neem oil, 200 ml methanol (Sigma-Aldrich) and 1.0 wt% KOH (Alfa Aesar). The experimental conditions were set at temperature of 333 K, experimental time of 3 h and stirring speed of 350 rpm. Finally, the resulting solution was allowed to settle for 24 h in order to separate the pure biodiesel and followed by washing with distilled water for several times. Water content in biodiesel was determined by coulometric Karl Fischer Titration (METTLER TOLEDO). Free and total glycerin in biodiesel was determined by gas chromatography (GC-2014, Shimadzu Corporation, Japan). Table 1 showed the physicochemical properties of the synthesized biodiesel^22,23^. The presence of water in the biodiesel was due to the synthesis process, which included washing the transesterification product.

**Table 1:** Specification of synthesized biodiesel from Neem oil.

Property	Unit	Biodiesel	ASTM Standard Biodiesel D 6751
Appearance	–	light Yellow	Light yellow
Odor	–	Mild	Mild
Physical State	–	Liquid	Liquid
Boiling point	°C	285	338 max
Kinematic Viscosity at 40 °C	mm^2^ s^-1^	5.07	1.9–6.0
Specific gravity at 25 °C	–	0.84	0.88
Flash point	°C	157	100–170
Pour point	°C	2.1	− 15 to 10
Water content	% vol	0.03	0.05 max
Free glycerin	wt%	0.018	0.02

### Methods

The weight loss WL was calculated by weighing before and after the immersion of the electrode in biodiesel for 1440 h using METTLER analytical balance. All the steps of WL were conducted according to ASTM G31-72(2004)^24^. The initial mass and area of the substrate were 7.4763 g and 5.734 cm^2^, respectively. The volume of biodiesel used was 100 ml. Three independent repeated experiments at the same conditions were carried out to ensure results validity. The resulted data were presented by the means and the standard deviation.

The EIS experiments were conducted in the standard cell (three electrodes: C-steel, saturated calomel electrode (SCE) reference electrode, Pt counter electrode) connected with electrochemical work-station (Gamry-3000)^25^. EIS curves were recorded in the frequency range of 30 kHz–1.0 Hz at open circuit potential using 20 mV amplitude**.**

The antioxidant test and TAN calculation for biodiesel at different conditions were carried out according to ASTM D943—20 and ASTM D664—18e2, respectively^26,27^.

The surface morphology (SEM and EDX) were conducted for C-steel samples in pure biodiesel and biodiesel containing 80 mg/l of [C_10_H_19_N_2_]^+^[C_2_F_6_NO_4_S_2_]^−^ by Scanning Electron Microscope SEM fitted with EDX analyzer (model: ZEISS/EVO, Carl Zeiss Microscopy).

## Results and discussion

### Anti-corrosion properties of [C_10_H_19_N_2_]^+^[C_2_F_6_NO_4_S_2_]^−^

To recognize the anti-corrosion properties of [C_10_H_19_N_2_]^+^[C_2_F_6_NO_4_S_2_]^−^, the WL and EIS methods were used for C-steel in biodiesel. The effect of [C_10_H_19_N_2_]^+^[C_2_F_6_NO_4_S_2_]^−^ on the rate of corrosion (ν) and anti-corrosion performance (*η*_w_%) of C-steel in biodiesel using the WL experiments is shown in Table 2.

**Table 2:** Weight loss parameters for the corrosion of C-steel in biodiesel and biodiesel containing [C_10_H_19_N_2_]^+^[C_2_F_6_NO_4_S_2_]^−^ at 298 K.

[C_10_H_19_N_2_]^+^[C_2_F_6_NO_4_S_2_]^−^ (mg/l)	ν (mg cm^-2^ h^-1^) × 10^–4^	*η*_w_ %
Blank (Biodiesel)	2.762 ± 0.135	–
20	1.692 ± 0.124	38.7
40	0.704 ± 0.035	74.5
60	0.235 ± 0.022	91.4
80	0.030 ± 0.002	98.9
100	0.033 ± 0.002	98.8
120	0.030 ± 0.002	98.9
140	0.049 ± 0.001	98.2

The ν and *η*_w_% were obtained using Eqs. (1) and (2)^28^:

1 $$\nu = \frac{W}{St}$$

2 $$\eta_{w} \% = \frac{{\nu_{0} - \nu }}{{\nu_{0} }} \times 100$$

W = C-steel weight loss, S = surface area, t = time of experiment, ν_0_ = corrosion rate in the blank solution.

[C_10_H_19_N_2_]^+^[C_2_F_6_NO_4_S_2_]^−^ inhibitor elicited a decrease in ν at 20 mg/l (from 2.762 × 10^–4^ to 1.692 × 10^–4^ mg cm^-2^ h^-1^), and this effect was sustained until the highest inhibitor concentration (i.e. 0.049 × 10^–4^ mg cm^-2^ h^-1^ at 120 mg/l) (see Table 2). Inhibition of corrosion activity of C-steel in biodiesel by [C_10_H_19_N_2_]^+^[C_2_F_6_NO_4_S_2_]^−^ was observed, with *η*_w_% values ranging from 38.7% to 98.9%. We noted that [C_10_H_19_N_2_]^+^[C_2_F_6_NO_4_S_2_]^−^ displayed the highest inhibition of 98.9% at 80 mg/l. Beyond concentration 80 mg/l, no significant change in the *η*_w_% values was observed. It appears that when 80 mg/l of ionic liquid was added, the ionic liquid molecules covered nearly all of the active centers on the C-steel, and that further addition had a limited impact on the inhibition efficiency. Similar observations were noted by Cao et al.^29^ and Arellanes-Lozada, et al.^30^.

The inhibition efficacy of [C_10_H_19_N_2_]^+^[C_2_F_6_NO_4_S_2_]^−^ in biodiesel medium compared to other inhibitors described in the literature (see Table 3)^31–35^.

**Table 3:** Inhibition efficacy of [C_10_H_19_N_2_]^+^[C_2_F_6_NO_4_S_2_]^−^ in biodiesel medium compared to other inhibitors described in the literature.

Compounds	Conc mg/l	Electrode	Solution	Efficiency %	Ref
N,N′-di-*sec*-butyl-p-phenylenediamine	500	Copper	Biodiesel obtained from babassu oil	87	^31^
Ethylenediamine	100	Carbon steel	Palm biodiesel	71.8	^32^
Vitex negundo leaf extract	2000	Aluminium	Biodiesel (B100) produced from waste cooking oil	83	^33^
Tert-butylamine	100	Cast iron	Biodiesel (B100)	49.41	^34^
Propyl gallate	400	Carbon steel	Biodiesel (B100)	83	^35^
[C_10_H_19_N_2_]^+^[C_2_F_6_NO_4_S_2_]^−^	80	Carbon steel	Biodiesel from Neem oil	98.9	This work

**Figure 1:**
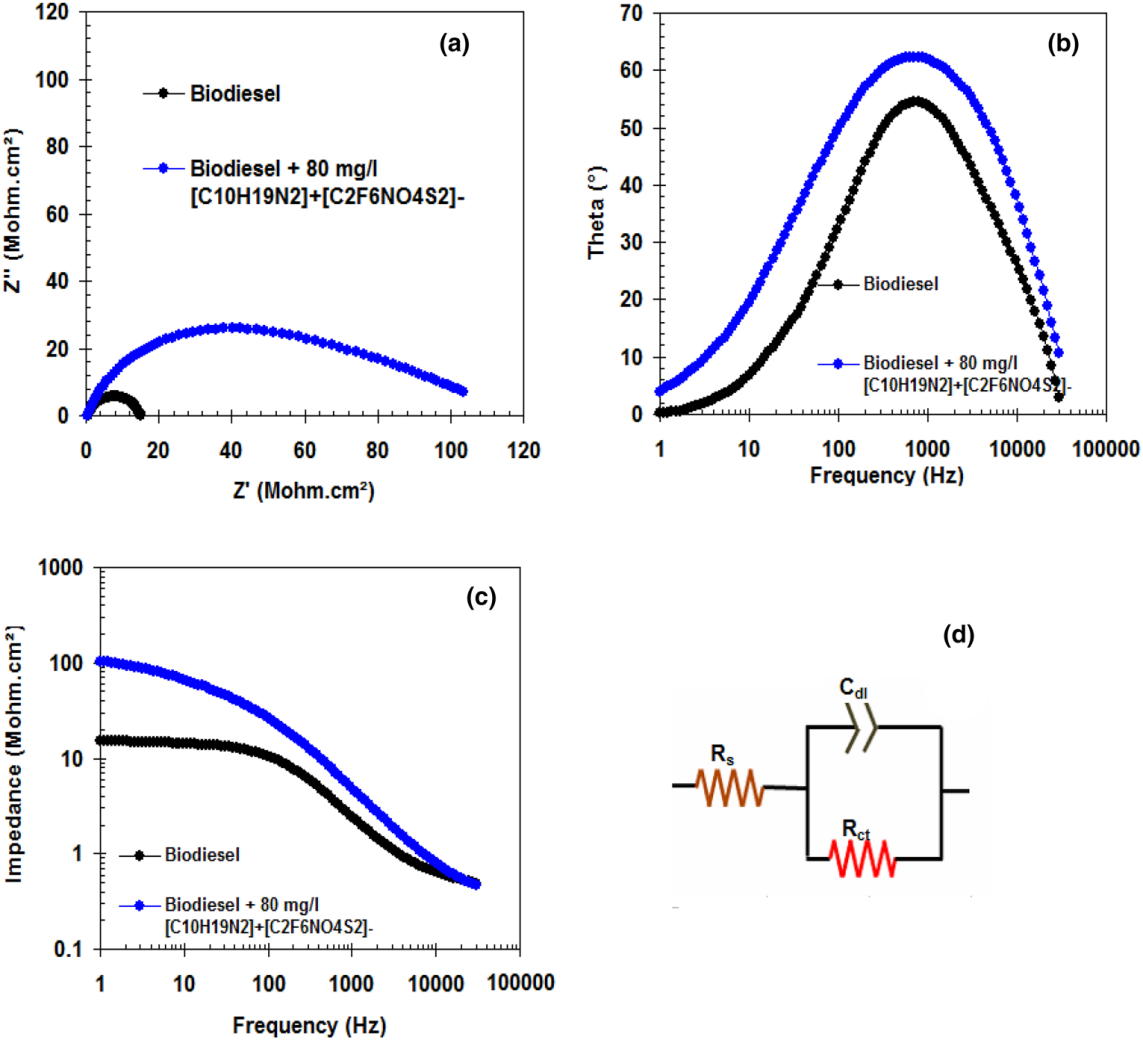
Typical EIS plots (a = Nyquist, b = Bode-phase angle, c = Bode-module, d = equivalent circuit) for the corrosion of C-steel in biodiesel and biodiesel containing 80 mg/l of [C_10_H_19_N_2_]^+^[C_2_F_6_NO_4_S_2_]^−^ at 298 K.

Further inspections on the performance of [C_10_H_19_N_2_]^+^[C_2_F_6_NO_4_S_2_]^−^ were conducted by using EIS measurements for C-steel in biodiesel without and with 80 mg/l of inhibitor. Typical EIS plots (a = Nyquist, b = Bode-phase angle, c = Bode-module, d = equivalent circuit) are shown in Fig. 1. The Nyquist plots (Fig. 1a) show one slightly depressed semicircle. Such non-ideal in the Nyquist plots is due to heterogeneity at the C-steel surface^36–38^. Two plateaus were visible in Fig. 1c, one at high frequency and the other at low frequency. The Nyquist plots, with and without [C_10_H_19_N_2_]^+^[C_2_F_6_NO_4_S_2_]^−^, can be described by Randles equivalent circuit (EC) as presented in Fig. 1d. In Fig. 1d, R_s_ is the solution resistance, C_dl_ is the double layer capacitor and R_ct_ is charge transfer resistance^39^. It is evident that for C-steel in biodiesel containing 80 mg/l of inhibitor, R_ct_ increased from 10.5 Mohm.cm^2^ (blank biodiesel) to 115.3 Mohm.cm^2^. Moreover, the addition of [C_10_H_19_N_2_]^+^[C_2_F_6_NO_4_S_2_]^−^ in biodiesel led to the decrease in the C_dl_ value from 1.51 nF cm^−2^ (blank biodiesel) to 0.13 nF cm^−2^. Additionally, the width of the Bode-phase angle (Fig. 1b) increases by adding [C_10_H_19_N_2_]^+^[C_2_F_6_NO_4_S_2_]^−^, which indicates a lower corrosion rate^40,41^. This means that [C_10_H_19_N_2_]^+^[C_2_F_6_NO_4_S_2_]^−^ is able to impede the corrosion of C-steel in biodiesel by forming a protective layer on the C-steel surface^42,43^.

### Thermodynamic activation and adsorption isotherms studies

**Figure 2:**
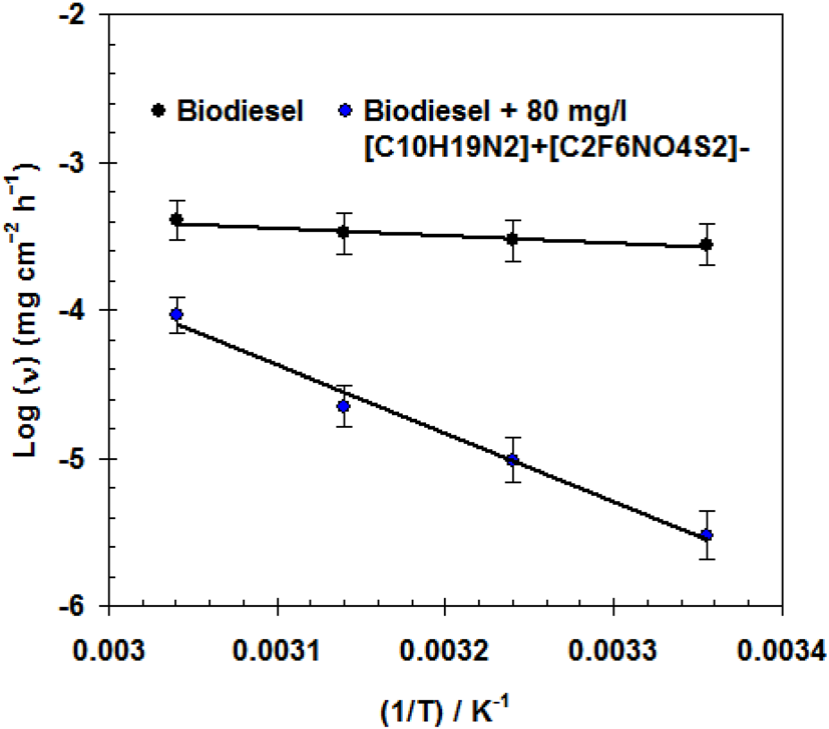
Arrhenius plot for C-steel in biodiesel without and with 80 mg/l of C_10_H_19_N_2_]^+^[C_2_F_6_NO_4_S_2_]^−^.

To estimate the performance of [C_10_H_19_N_2_]^+^[C_2_F_6_NO_4_S_2_]^−^ at high temperatures circumstances, the ν and *η*_w_% values for C-steel in biodiesel without and with 80 mg/l of inhibitor were calculated in the range 298–328 K. It was noted that, under an elevated temperature of 298 K to 328 K, the *η*_w_% value slightly decreases from 98.9 to 91.9% and the corrosion rate increases from (0.030 ± 0.002) × 10^–4^ to (0.396 ± 0.010) × 10^–4^ mg cm^−2^ h^−1^^44^ (see Fig. 2). This indicates that [C_10_H_19_N_2_]^+^[C_2_F_6_NO_4_S_2_]^−^ retains its performance at high temperature, confirming its thermal stability^45^.

To assess the activation energy (*E*_a_) for C-steel in biodiesel without and with 80 mg/l of [C_10_H_19_N_2_]^+^[C_2_F_6_NO_4_S_2_]^−^, the variation of log (ν) with (1/*T*) was plotted , as displayed in Fig. 2, according to Arrhenius formula (Eq. 3)^46,47^.

3 $$\nu = \lambda e^{{\frac{{ - E_{a} }}{RT}}}$$

The *E*_a_ for C-steel in blank biodiesel was 4.17 kJ mol^-1^. Whereas, with the addition of the 80 mg/l of [C_10_H_19_N_2_]^+^[C_2_F_6_NO_4_S_2_]^−^, the value increased to 38.08 kJ mol^-1^. This refers to the strong physical adsorption of [C_10_H_19_N_2_]^+^[C_2_F_6_NO_4_S_2_]^−^ on the C-steel surface^48,49^. Where the ionic liquid molecules create a large energy barrier against the corrosion process of C-steel in biodiesel^50^.

The adsorption isotherm models that describe the adsorption of [C_10_H_19_N_2_]^+^[C_2_F_6_NO_4_S_2_]^−^ on the C-steel surface based on the WL measurements were inspected.

To choose the best isotherm for the current case, various adsorption isotherm models such as Langmuir, Freundlich, and Temkin were tested (Eqs. 4, 5 and 6).

4 $$\frac{{C_{inh} }}{\theta } = \frac{1}{{K_{{{\text{ads}}}} }} + C_{inh} \quad {\text{Langmuir}}$$

5 $$\theta = K_{{{\text{ads}}}} \left( {C_{{{\text{inh}}}} } \right)^{{{1}/n}} \quad {\text{Freundlich}}$$

6 $${\text{exp}}( - {\text{2a}}\theta ) \, = K_{{{\text{ads}}}} C_{{{\text{inh}}}} \quad {\text{Temkin}}$$

where *C*_inh_ is the ionic liquid concentration, *K*_ads_ is the equilibrium constant, “a” is the molecules interaction parameter, and *θ* is the surface coverage = *η*_w_ %/ 100.

**Figure 3:**
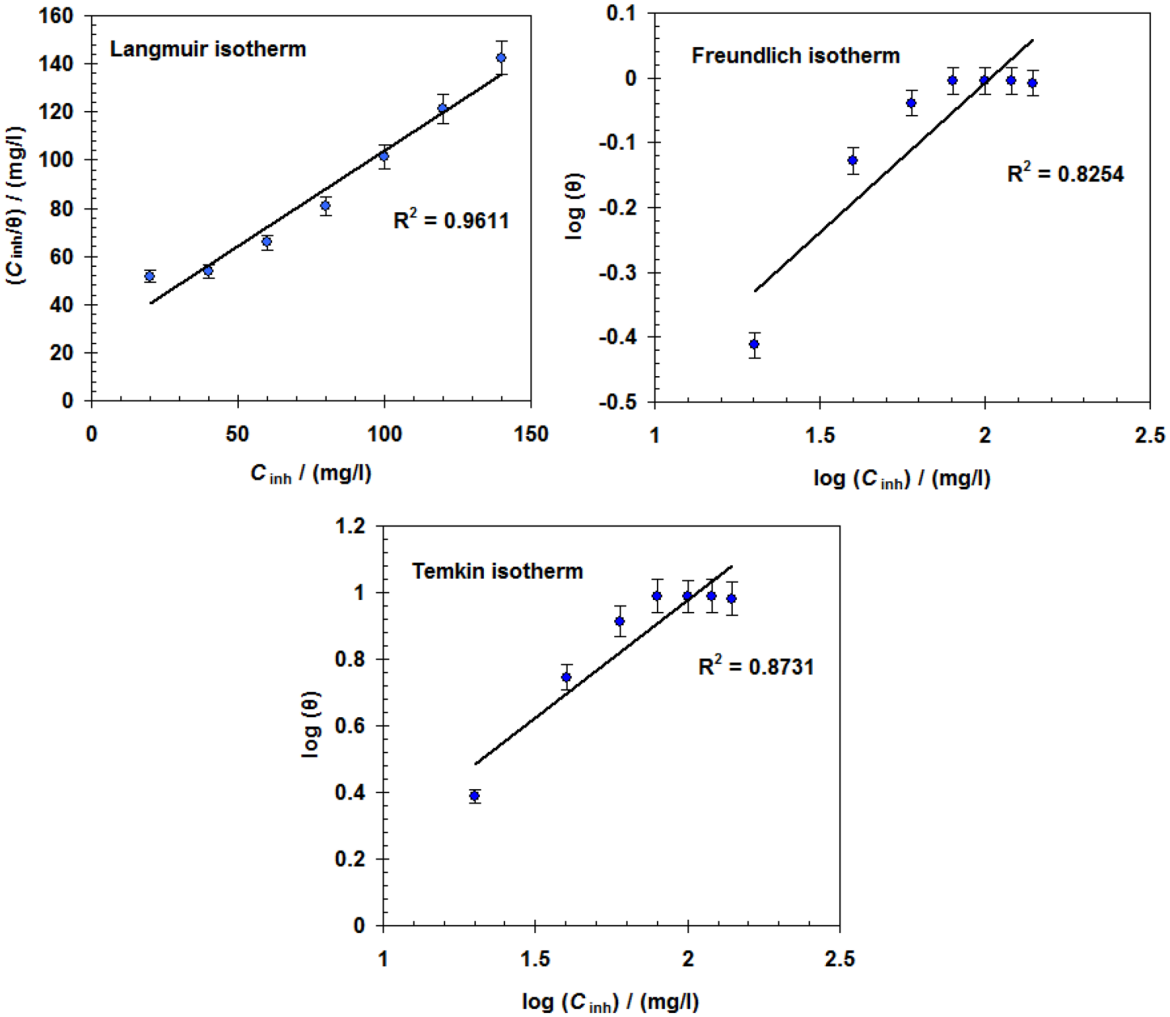
Various adsorption isotherm plots for [C_10_H_19_N_2_]^+^[C_2_F_6_NO_4_S_2_]^−^ adsorption on C-steel surface in biodiesel at 298 K.

According to the data in Fig. 3, the Langmuir adsorption isotherm is the best isotherm for this case. This is dependent on the correlation coefficient (R^2^) being close to unity ^51^.

The *K*_ads_ value for the [C_10_H_19_N_2_]^+^[C_2_F_6_NO_4_S_2_]^−^ is about 1.80 × 10^4^ M^-1^.

Moreover, the Eq. 7 can be utilized to calculate the standard free energy of the adsorption reaction (∆G°_ads_) ^52^.

7 $$\Delta {\text{G}}_{{{\text{ads}}}}^{^\circ } = \, - RT{\text{ln }}\left( {{55}.{5}K_{{{\text{ads}}}} } \right)$$

The ∆G°_ads_ for the [C_10_H_19_N_2_]^+^[C_2_F_6_NO_4_S_2_]^−^ is about—34.16 kJ mol^−1^. The negative value of ∆G°_ads_ clarified the spontaneous adsorption of [C_10_H_19_N_2_]^+^[C_2_F_6_NO_4_S_2_]^−^ molecules on the C-steel surface^53^. Because the value of ∆G°_ads_ is less than—40 kJ mol^−1^, the type of adsorption may be physisorption or mixed type (physisorption and chemisorption)^54^.

### SEM and EDX analysis

**Figure 4:**
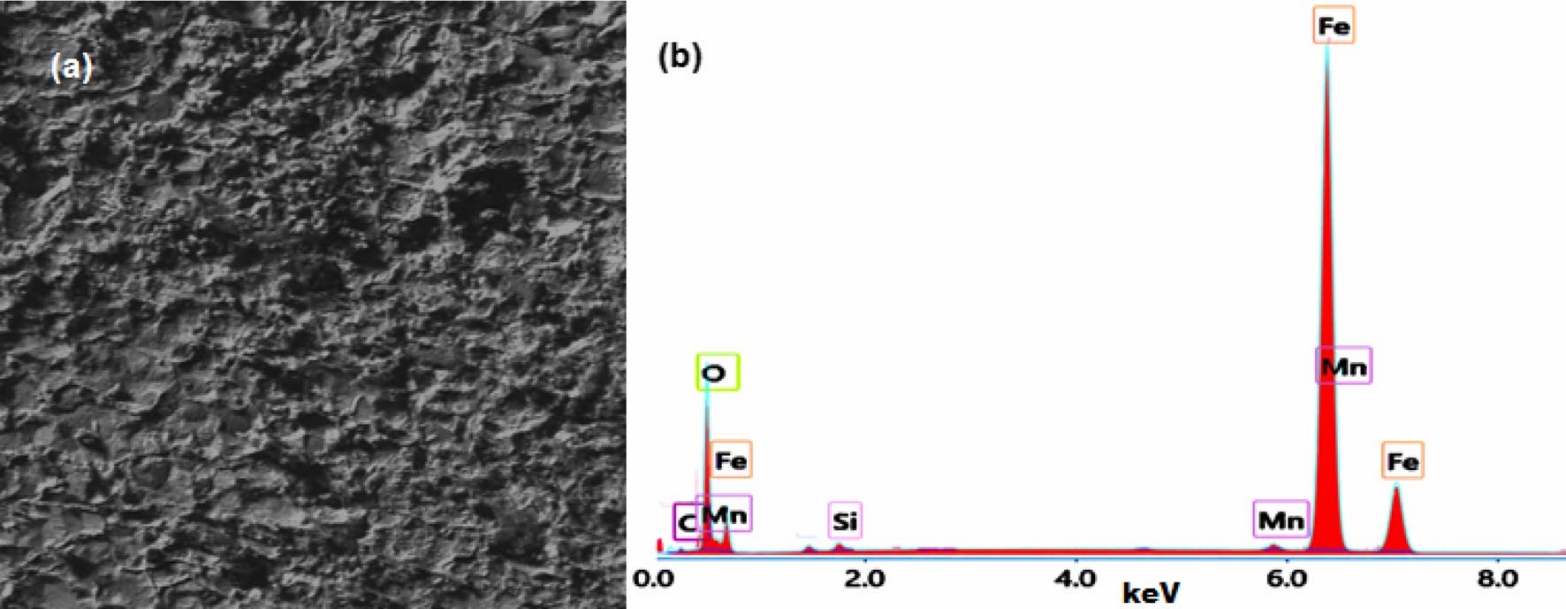
SEM image (**a**) and EDX analysis (**b**) of C-steel in biodiesel.

**Figure 5:**
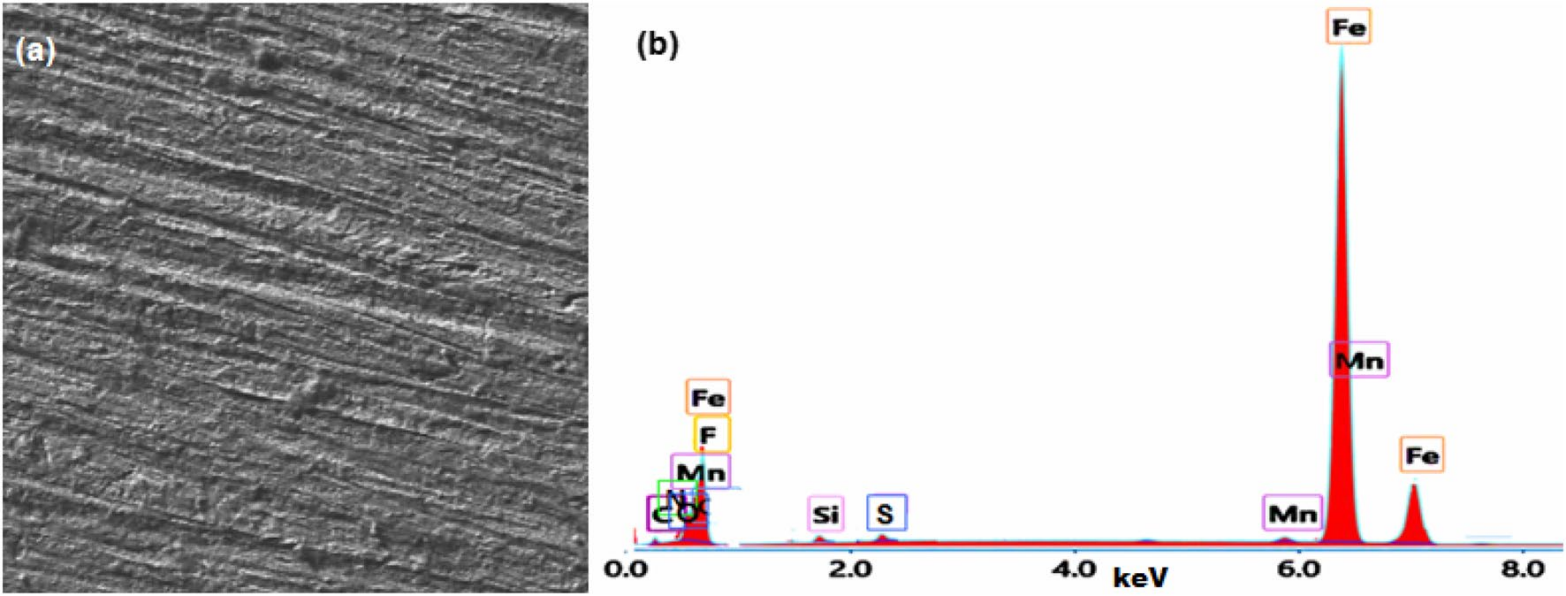
SEM image (**a**) and EDX analysis (**b**) of C-steel in biodiesel containing 80 mg/l of [C_10_H_19_N_2_]^+^[C_2_F_6_NO_4_S_2_]^−^.

The SEM and EDX analysis of C-steel in biodiesel without and with 80 mg/l of [C_10_H_19_N_2_]^+^[C_2_F_6_NO_4_S_2_]^−^ are shown in Figs. 4 and 5. The C-steel surface, immersed in biodiesel for 1440 h, without [C_10_H_19_N_2_]^+^[C_2_F_6_NO_4_S_2_]^−^ was extremely damaged due to the aggressive medium (Fig. 4a). EDX analysis for this case (Fig. 4b), reveals the signals for C-steel composition (i.e. C, Si, Mn, Fe) and corrosion products (i.e. iron oxide).

The impact of adding 80 mg/l of [C_10_H_19_N_2_]^+^[C_2_F_6_NO_4_S_2_]^−^ to the biodiesel on the C-steel surface is shown in Fig. 5a. It is clear that the surface of C-steel is smooth and no corrosion products were observed on the metal surface. EDX analysis for this case (Fig. 5b), reveals the signals for C-steel composition (i.e. C, Si, Mn, Fe) and characterized signals of [C_10_H_19_N_2_]^+^[C_2_F_6_NO_4_S_2_]^−^ (i.e. C, N, F,O, S).

### Mechanism of corrosion mitigation

Corrosion mitigation of C-steel in biodiesel using ionic liquid [C_10_H_19_N_2_]^+^[C_2_F_6_NO_4_S_2_]^−^ is related to two factors. The first is the adsorption ability of [C_10_H_19_N_2_]^+^[C_2_F_6_NO_4_S_2_]^−^ molecules on the C-steel surface to form a shielding layer^55,56^. This layer can isolate the C-steel surface from the biodiesel^57^. The presence of hetero-atoms (O, S, and N atoms) in the ionic liquid molecule affects the efficiency of this inhibitor. These atoms are commonly regarded as the reaction centre for initiating the adsorption process^58–60^. The nonbonding electrons present on hetero-atoms, as well as π-electrons, will be transferred into the d-orbitals of the Fe atoms on the steel surface, leading to the formation of coordinate bonds between C-steel and the adsorbed ionic liquid, as observed for many organic inhibitors^61,62^. SEM and EDX analysis verified the ionic liquid's adsorption on the C-steel surface, as shown in Figs. 4 and 5.

**Figure 6:**
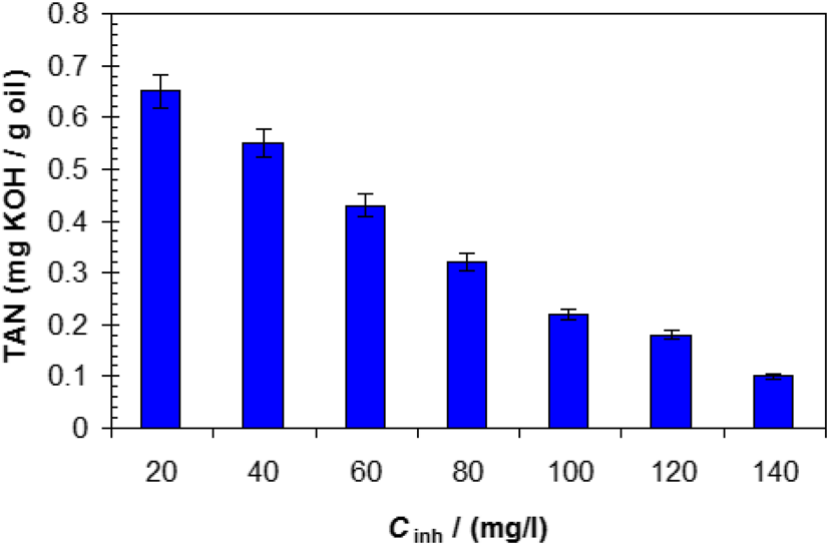
Variation of TAN of biodiesel with C_10_H_19_N_2_]^+^[C_2_F_6_NO_4_S_2_]^−^ concentration.

The second factor is the antioxidant properties of ionic liquid^63^. This leads to the decrease in the oxidation of biodiesel and consequently, prevents the formation corrosion compounds such as free acids and aldehydes^16^. To confirm antioxidant properties of [C_10_H_19_N_2_]^+^[C_2_F_6_NO_4_S_2_]^−^, the impact of adding different concentrations of C_10_H_19_N_2_]^+^[C_2_F_6_NO_4_S_2_]^−^ on the TAN of biodiesel was recorded and shown in Fig. 6. Inspection of Fig. 6 confirms that the presence of [C_10_H_19_N_2_]^+^[C_2_F_6_NO_4_S_2_]^−^ leads to low TAN of biodiesel. This decreases the corrosive action of biodesel especially during the long time storage.

## Conclusions

The necessity to control corrosion in fuel tanks containing biodiesel motivated us to explore the anti-corrosion properties of ionic liquid [C_10_H_19_N_2_]^+^[C_2_F_6_NO_4_S_2_]^−^, that could serve as informative to control the corrosion of C-steel in biodiesel. [C_10_H_19_N_2_]^+^[C_2_F_6_NO_4_S_2_]^−^, reveals an effective new C-steel corrosion inhibitor in biodiesel. The inhibition mechanism is based on the ionic liquid's mixed physisorption and chemisorption. [C_10_H_19_N_2_]^+^[C_2_F_6_NO_4_S_2_]^−^ molecules cover the surface of C-steel sheets, preventing biodiesel corrosive attack on steel sites. The inhibition effect is explained by this protective layer and the adsorption of an ionic liquid compound. It was clear that the [C_10_H_19_N_2_]^+^[C_2_F_6_NO_4_S_2_]^−^ displayed the highest inhibition 98.9% at 80 mg/l. The Nyquist and Bode plots conclude that the inhibition effect improves with increasing [C_10_H_19_N_2_]^+^[C_2_F_6_NO_4_S_2_]^−^ concentration: the charge transfer resistance increases significantly while the capacitance of the electrical double layer decreases dramatically. The studies of the impact of [C_10_H_19_N_2_]^+^[C_2_F_6_NO_4_S_2_]^−^ concentration and temperature allows for the determination of thermodynamic parameters and the confirmation of the protective role of the ionic liquid layer. The antioxidant properties of [C_10_H_19_N_2_]^+^[C_2_F_6_NO_4_S_2_]^−^ play a significant role in explaining the anti-corrosion mechanism.
